# Symptom Recognition as a Mediator in the Self-Care of Chronic Illness

**DOI:** 10.3389/fpubh.2022.883299

**Published:** 2022-05-17

**Authors:** Barbara Riegel, Maddalena De Maria, Claudio Barbaranelli, Maria Matarese, Davide Ausili, Anna Stromberg, Ercole Vellone, Tiny Jaarsma

**Affiliations:** ^1^School of Nursing, University of Pennsylvania, Philadelphia, PA, United States; ^2^Department of Biomedicine and Prevention, University of Rome Tor Vergata, Rome, Italy; ^3^Department of Psychology, Faculty of Medicine and Psychology, Sapienza University of Rome, Rome, Italy; ^4^Research Unit of Nursing Sciences, Campus Bio-Medico University of Rome, Rome, Italy; ^5^Department of Medicine and Surgery, University of Milan-Bicocca, Monza, Italy; ^6^Department of Health, Medicine and Caring Sciences, Department of Cardiology, Linkoping University, Linköping, Sweden; ^7^Department of Health, Medicine and Caring Sciences, Linkoping University, Linköping, Sweden

**Keywords:** self-care, self-management, chronic illness, chronic disease, symptom perception, interoception, mediation analysis, symptom recognition

## Abstract

**Background:**

The recognition of a symptom is needed to initiate a decision to engage in a behavior to ameliorate the symptom. Yet, a surprising number of individuals fail to detect symptoms and delay in addressing early warnings of a health problem.

**Purpose:**

The aim of this study was to test the hypothesis that symptom recognition mediates the relationship between monitoring for and management of symptoms of a chronic illness.

**Methods:**

A secondary analysis of existing cross-sectional data. A sample of 1,629 patients diagnosed with one or more chronic conditions was enrolled in the United States (US) (*n* = 407), Italy (*n* = 784) and Sweden (*n* = 438) between March 2015 and May 2019. Data on self-care monitoring, symptom recognition, and self-care management was assessed using the Self-Care of Chronic Illness Inventory. After confirming metric invariance in cultural assessment, we used structural equation modeling to test a mediation model where symptom recognition was conceptualized as the mediator linking self-care monitoring and self-care management with autonomous (e.g., Change your activity level) and consulting behaviors (e.g., Call your healthcare provider for guidance).

**Results:**

Symptom recognition mediated the relation between self-care monitoring and autonomous self-care management behaviors (β = 0.098, β = 0.122, β = 0.081, *p* < 0.001 for US, Italy, and Sweden, respectively). No mediation effect was found for consulting self-care management behaviors.

**Conclusion:**

Our findings suggests that symptom recognition promotes autonomous self-care behaviors in people with a chronic condition. Self-care monitoring directly affects consulting self-care management behaviors but not through symptom recognition. Further research is needed to fully understand the role of symptom recognition in the self-care process.

## Introduction

Symptom recognition is the cue used by patients to indicate the need for a self-care response (1–6). Symptom recognition involves detection and interpretation. Yet, there is growing awareness that some patient populations experience impaired ability to detect and interpret symptoms. The insular cortex in the brain is associated with symptom perception (7), and some people with heart failure (HF), diabetes mellitus (DM), stroke, and other conditions have been shown to have damage to the insular cortex (8, 9). We have been studying self-care in people with chronic illness as a process of maintaining health, monitoring symptoms, and managing symptoms when they occur (10). Symptom recognition is a fundamental element of this work but the growing literature on interoception led us to question whether symptom recognition is a self-care behavior or a physiologic phenomenon.

Self-care is defined as a process of maintaining health through health promoting practices and managing illness when it occurs (10). Theoretically this process involves three linked sequential behaviors captured in the key concepts of self-care maintenance, self-care monitoring, and self-care management (10). Self-care maintenance addresses behaviors used by patients with a chronic illness to maintain physical and emotional stability (e.g., medication adherence), while self-care monitoring involves the behavior of observing oneself for signs and symptoms (e.g., checking blood pressure). We have argued that self-care monitoring is the link or bridge between self-care maintenance and self-care management (11). A core goal of self-care monitoring is symptom recognition; once recognized, self-care management (e.g., take a medicine for a symptom) can take place, with behaviors that reflect a response to the symptoms observed.

### Symptom Recognition as a Physiological Process

**Figure 1:**
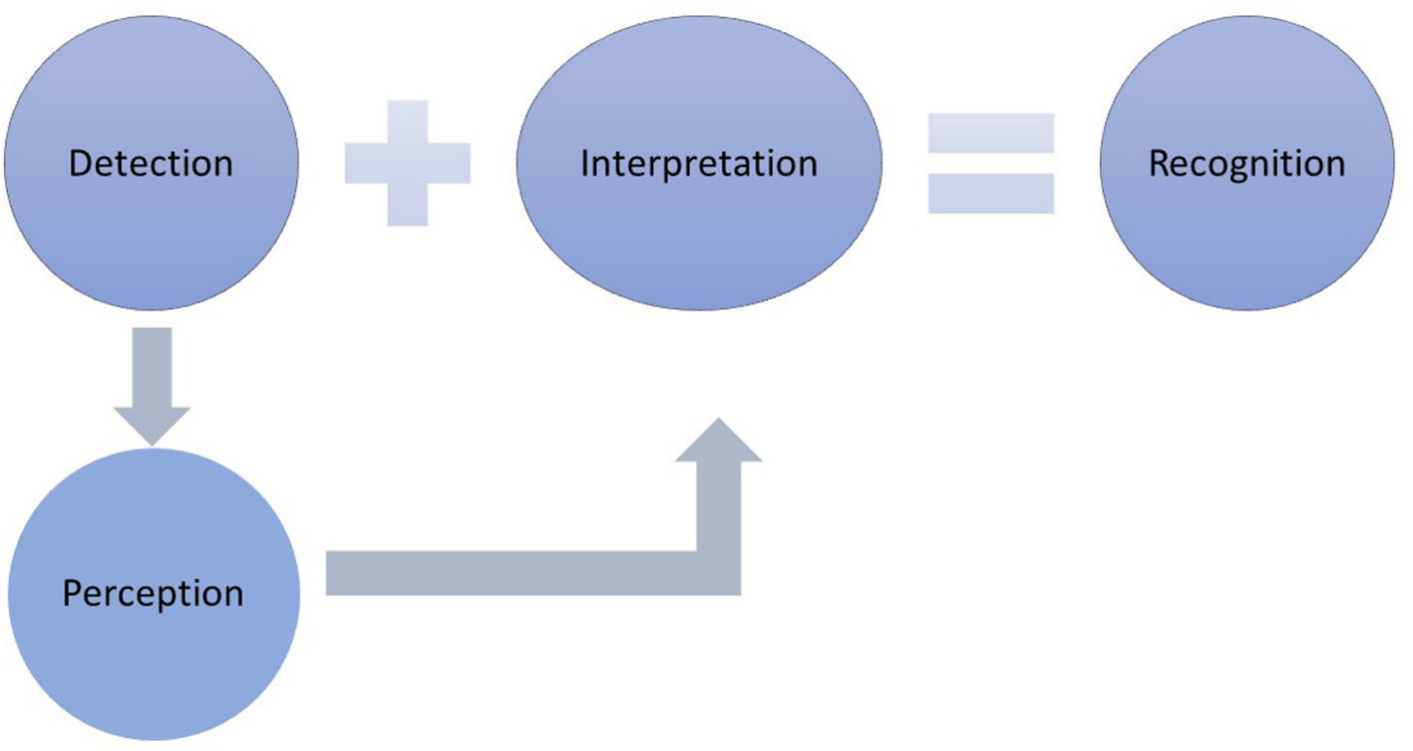
The response to symptoms requires that the symptom is detected, presumably through the behaviors of self-care monitoring. Monitoring involves actively noticing typical symptoms and checking for the presence of bodily changes. Symptom detection precedes perception, which involves becoming consciously aware of the symptom. Once detected and perceived, interpretation and the assignment of meaning occurs, which leads to symptom recognition. Symptom recognition is required before a self-care management response can be expected to occur.

Interoception is defined as the sense of the internal body, reflecting the processes by which one senses, interprets, integrates, and regulates internal signals (9, 12). Interoception provides information about what is happening inside the body (e.g., hunger, thirst, racing heart, sexual arousal). As such, interoception appears to be an essential stimulus of behavior, one that is necessary but not sufficient to generate a response. That is, after one detects what is happening in the body, the feeling is perceived as it comes to consciousness and progresses to recognition if the feeling is interpreted and assigned meaning (Figure 1). This interpretation is required before a behavioral response is generated (13). There is accumulating evidence that interoception influences the decisions made about symptoms (14).

As an example, consider individuals with type 1 DM who live with unpredictable hypoglycemia. Recognition of the symptoms of hypoglycemia (e.g., feeling shaky, nervous, irritable, confused, hungry, dizzy) is the primary defense against severe hypoglycemia. Yet, as many as 60% of individuals with type 1 DM experience impaired awareness of hypoglycemia (15). In patients with type 1 DM hypoglycemia unawareness, the brain response to hypoglycemia is blunted (16). Regional brain defects have been found in the insula of patients with type 2 DM (17) and it may be that a lack of symptom perception and recognition in DM are associated with these defects. Without the perception of relevant symptoms, the person with type 1 DM cannot be expected to recognize hypoglycemia and decide to engage in self-care.

Damage in other areas of the brain affects self-care as well. When there is damage to the frontal cortex, there is an adverse impact on decision-making, conflict resolution, and memory (18). When decision-making is impaired, patients recognize that they have a symptom but are unsure if it is important. Problems with conflict resolution occur when patients are unable to make up their minds if given a choice on what to do, an issue associated with right cingulate cortex injury (18). Memory problems or inability to remember if the symptom is important and what they are supposed to do about it may reflect hippocampal injury (19, 20). Thus, multiple areas of brain injury can dramatically impact self-care ability, not just in symptom perception, but also in terms of deciding if the physical/body change is important, whether or not they should do something about it, and remembering what they were told to do.

As symptom recognition is not a behavior but a signal to engage in behavior such as self-care, we have struggled to identify the best place for this concept in the theory. In our original theoretical article, we wrote that self-care management involves an evaluation of changes in physical and emotional symptoms (10). We also proposed that monitoring for changes in signs or symptoms is necessary for effective self-care management because one cannot decide what to do about a symptom unless it has been noticed and evaluated. In an update to the theory we discussed symptom recognition as part of self-care monitoring (11). As described further below, in psychometric testing it was unclear where the item assessing symptom recognition should be placed (21, 22). Together these observations led us to propose that symptom recognition may be a distinct concept separate from self-care monitoring and self-care management. The aim of this study was to test the hypothesis that symptom recognition is a mediator of the relationship between self-care monitoring and self-care management, two core concepts in the Theory of Self-Care of Chronic Illness (10). Identifying symptom recognition as a mediator would confirm our tentative observation that symptom recognition is neither a component of self-care monitoring nor self-care management.

## Materials and Methods

### Design

The method used to test this hypothesis was a secondary analysis of existing cross-sectional data.

### Sample/Participants

The US sample included 407 participants from inpatient and outpatient settings in the eastern and southern US in addition to a small sample from ResearchMatch.org, a national electronic, web-based registry of research volunteers. Participants were required to be 18 years of age or older and able to read and write in English. All had at least one chronic illness, but no effort was made to choose specific illnesses. Persons with a diagnosis of dementia were excluded.

The Italian sample included 784 participants from three different studies. The first sample was from a multicenter cross-sectional study aimed at measuring the psychometrics properties of the SC-CII in outpatients and inpatients aged ≥ 18 years from Southern and Central Italy. All study participants had to have heart failure (HF), chronic obstructive pulmonary disease (COPD), type 1 or type 2 DM, or Parkinson's disease. Patients with a diagnosis of dementia were excluded. The second sample was enrolled in a cross-sectional study conducted to evaluate outcomes associated with self-care in adult (≥18 years) outpatients with type 2 DM recruited in Northern Italy. Patients with cognitive impairment and illiteracy were excluded. The third sample was from the SODALITY study, a longitudinal investigation aimed at evaluating self-care in inpatients and outpatients aged ≥ 65 years and their family caregivers (23). These patients were enrolled in Southern and Central Italy if they had a diagnosis of DM, COPD, or HF and at least one other chronic condition. Patients with a diagnosis of cancer or dementia were excluded.

The Swedish sample was drawn from a cross-sectional study evaluating continuity of care and self-care with a cardiac diagnosis (HF, arrhythmia, angina, myocardial infarction) following an unplanned hospitalization. The full sample of more than 1,000 participants was enrolled consecutively from four hospitals. In the present study, a subsample of 438 adults was chosen if they had at least one comorbid illness in addition to the primary cardiac diagnosis. Any patient with dementia or inability to read and write in Swedish was excluded. Study participants were mailed a survey packet, which included the SC-CII 6–8 weeks after hospital discharge.

The samples from the three countries were compared in the publication describing the psychometric analysis of the SC-CII (22). All three samples included adults over age 62 years on average. All participants had two or more chronic conditions or multimorbidity. At least one third of each sample was female. The Italian sample had a lower level of education (74.9% with less than a high school education vs. 10.8% of the US sample). More than half of all participants were married. Most were retired. Data were collected between March 2015 and May 2019.

### Ethical Considerations

Before data collection, the original study protocols underwent ethical approval in the US, Italy, and Sweden. All participants were fully informed of the aims of the studies and gave their written informed consent. Participants were assured that their data would be kept confidential, and they could withdraw from the study at any time. All the analyses performed in this study were performed after de-identifying each dataset.

### Measurement

Data on self-care was obtained using the Self-Care of Chronic Illness Inventory (SC-CII), a 20-item self-report instrument measuring the core processes of self-care maintenance, self-care monitoring, and self-care management using three separate scales: Self-Care Maintenance, Self-Care Monitoring, and Self-Care Management (21). The Self-Care Maintenance scale measures behaviors performed to maintain health with two dimensions: health promoting behavior and consulting behavior. Examples of self-care maintenance items are *Make sure to get enough sleep* (a health promoting behavior) and *Take prescribed medicines without missing a dose* (a consulting behavior). An example of a Self-Care Monitoring item includes *Monitor for symptoms*. The Self-Care Management scale measures the behavioral responses to symptoms with two dimensions: autonomous and consulting behaviors. Autonomous behaviors (i.e., *Change what you eat or drink to make the symptom decrease or go away, Change your activity*) are implemented directly by the patient. Consulting behaviors are based on guidance from healthcare providers (i.e., *Take a medicine to make the symptom decrease or go away, Tell your healthcare provider about the symptom at the next office visit, Call your healthcare provider for guidance*). The SC-CII measures self-care as a generic phenomenon applicable to a wide variety of conditions. That is, most of the self-care behaviors addressed in the instrument (e.g., get enough sleep, eat a healthy diet) are applicable to any illness. Each of the three scales—Self-Care Maintenance, Self-Care Monitoring, and Self-Care Management—is standardized mathematically to yield a score of 0–100. A cut-point of 70 or greater on each scale is used to judge self-care adequacy (24). An 8-point difference in standardized scale scores is considered a minimally important difference in scores (25). Composite reliability coefficients, computed for this study using data from each country, ranged from 0.69 to 0.86 for the three scales.

In the Self-Care of Chronic Illness Inventory (SC-CII) (21), a single item is used to assess symptom recognition (item #14: *The last time you had symptoms, how quickly did you recognize it as a symptom of your illness?*). Response choices include not applicable for people without symptoms, 0 (I did not recognize the symptom), or 1–5 (not quickly to very quickly). In psychometric testing item #14 performed inconsistently. Specifically, item #14 failed to load on the Self-Care Monitoring scale, where we expected it to fit. Instead, it loaded on the autonomous behavior subscale of the Self-Care Management scale but only after we set the cross loading of the item at 0 (21). In this study we analyzed item #14 separately, not within either the Self-Care Monitoring scale or the Self-Care Management scale. For the analysis we used the 5-point ordinal response scale (1 “not quickly” to 5 “very quickly”) to form an observed variable for use in the mediation analysis.

### Data Analysis

Analysis began with a cross-cultural assessment of scale dimensionality. Specifically, measurement equivalence (ME), or measurement invariance (MI), was used to determine whether the interpretation of the measured construct was conceptually similar in the different groups (26). We used the framework developed by Meredith (1993) to test ME, implemented via Multiple Group Confirmatory Factor Analysis (MG-CFA). This approach allowed us to simultaneously test a unique model on two or more samples maintaining the specificity of each sample. Increasingly stringent equality constraints were positioned in a series of nested models (27) to test for the invariance/equivalence of a same parameter (e.g., a regression coefficient linking two focal constructs) across samples. Partial scalar invariance levels were reached for the Self-Care Maintenance, Self-Care Monitoring, and Self-Care Management scales of the SC-CII (22). In this study, we evaluated the possibility of excluding item #14 from the Self-Care Management scale for the theoretical reasons described above. Thus, we replicated the same analysis we performed in the previous study with the same data sets (22).

In the second step, using Maximum Likelihood robust (MLr) estimation, we performed a mediation multi-group analysis to evaluate the mediating effect of item #14 in the relationship between Self-Care Monitoring and the two subscales of the Self-Care Management scale (autonomous and consulting behaviors) using data from all three countries. This analysis was performed using full structural equation modeling (SEM). To evaluate model fit, we used several goodness-of-fit indices: Comparative Fit Index (CFI), Tucker and Lewis Index (TLI), Root Mean Square Error of Approximation (RMSEA), and Standardized Root Mean Square Residual (SRMR) (28–30). CFI and TLI were used to compare the model of interest with a null model (31), with values of 0.90–0.95 indicating acceptable fit and values > 0.95 indicating good fit (32). RMSEA was used to estimate the lack of model fit, with values of ≤ 0.05 indicating a well-fitting model, 0.05–0.08 indicating a moderate fit, and ≥0.10 indicating poor fit (33). SRMR is a measure of fit in the sample, with values ≤ 0.08 indicating a good fit. Traditional chi-square (χ^2^) statistics were used in interpreting model fit.

Finally, to evaluate if the same effects of Self-Care Monitoring on autonomous and consulting behaviors via item #14 were present among the three countries, we posited equality constraints. Standardized and unstandardized coefficients are reported to present the effect among variables. Full Information Maximum Likelihood (FIML) method was used to estimate missing data values (34). A *p*-value <0.05 was considered statistically significant. All analyses were performed with Mplus program version 8.4.

## Results

**Figure 2:**
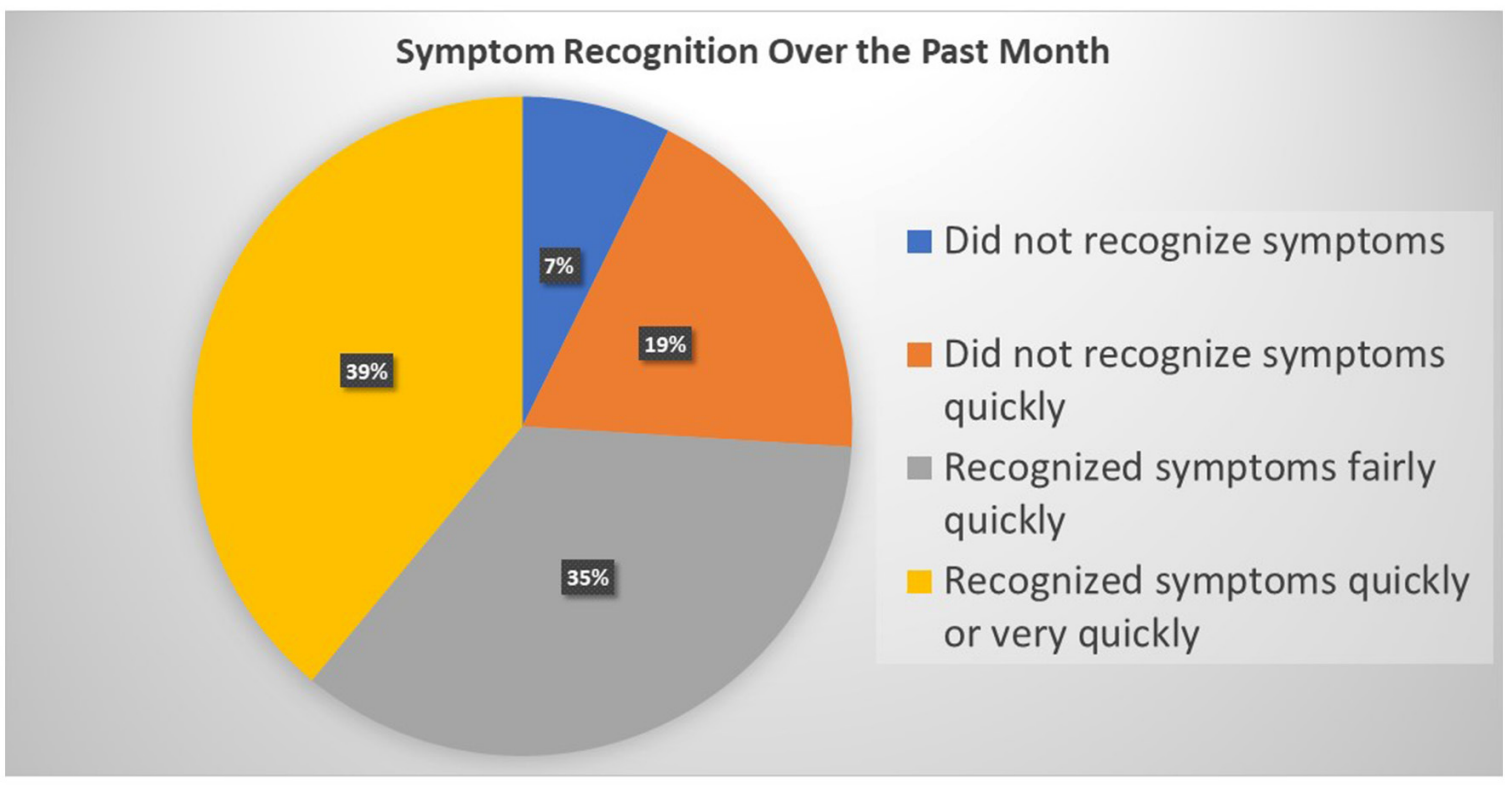
Graphic illustration of the way responses to this question can be analyzed.

Patients reported primarily hypertension (79.7%), a cardiac diagnosis (72.1%), DM (42.1%), chronic kidney failure (11.5%), and COPD (9.0%). A large proportion of the sample reported not experiencing symptoms in the past month (42.5%). Of those who experienced symptoms, 7.3% (*n* = 68) did not recognize their symptom. Of those who recognized the symptom when it occurred, the rapidity of recognition differed markedly (Figure 2).

In comparing the data across countries, metric invariance level was reached for Self-Care Monitoring and Self-Care Management [$\chi_{\left(163,\text{N}=1,615\right)}^{2}$ = 275.294, *p* < 0.001, CFI = 0.968, TLI = 0.961, RMSEA = 0.036 (90% CI = 0.028 0.043), *p* = 0.860, SRMR = 0.060 (first panel of Table 1)]. Metric invariance is the level required for a meaningful comparison of regression coefficients (i.e., the “betas”) across countries. Self-Care Maintenance was not tested because it was not used in the analysis.

**Table 1:** Parameter estimates from the final solution of the MG-CFA for testing measurement invariance, and from the mediational model tested on the US, Italian, and Swedish samples (*n* = 1,615).

**Results from measurement invariance analysis**
**Self-care monitoring**	**US**	**Italy**	**Sweden**
Listed below are common things that people with chronic illness monitor. How often do you do the following?			
9. Monitor your condition?	0.589	0.576	0.648
10. Pay attention to changes in how you feel?	0.717	0.642	0.566
11. Monitor for medication side-effects?	0.731	0.656	0.685
12. Monitor whether you tire more than usual doing normal activities?	0.742	0.682	0.774
13. Monitor for symptoms?	0.869	0.775	0.860
**Self-care management**	**US**		**Italy**		**Sweden**	
When you have symptoms, how likely are you to …
15. Change what you eat or drink to make the symptom decrease or go away?	0.615	0	0.484	0	0.583	0
16. Change your activity level (e.g., slow down, rest)?	0.578	0	0.425	0	0.692	0
20. Think of a treatment you used the last time you had symptoms. Did the treatment you used make you feel better?	0.591	0	0.538	0	0.559	0
17. Take a medicine to make the symptom decrease or go away?	0.318	0	0.249	0	0.598*	0
18. Tell your healthcare provider about the symptom at the next office visit?	0	0.805	0	0.800		0.776
19. Call your healthcare provider for guidance?	0	0.605	0	0.639		0.641
**Results from mediational model**
**Self-care monitoring** ** → symptom recognition** ** → consultive self-care management behaviors**
	**b**	**β**	**SE**	***p*** **-value**
US	−0.011	−0.009	0.017	0.596
Italy	−0.011	−0.008	0.015	0.597
Sweden	−0.011	−0.007	0.014	0.597
**Self-care monitoring** ** → symptom recognition** ** → autonomous self-care management behaviors**
	**b**	**β**	**SE**	***p*** **-value**
US	0.125	0.098	0.017	<0.001
Italy	0.125	0.122	0.020	<0.001
Sweden	0.125	0.081	0.014	<0.001

*b, unstandardized coefficients; β, standardized coefficients; SE, Standard Error*.

The loading estimates come from the completely standardized solution. Since constraints are imposed on the unstandardized estimates, factor loadings differ only apparently across the three countries. All loadings are invariant except where noted by *.

**Figure 3:**
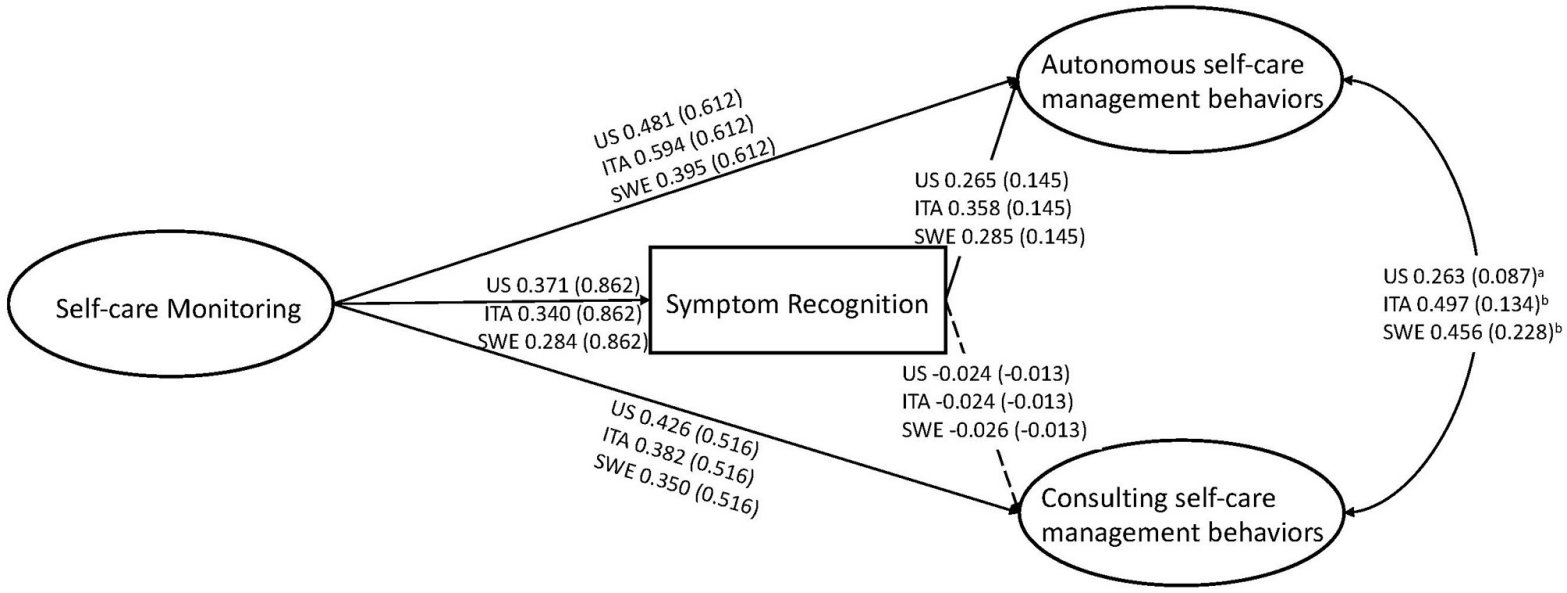
Direct effects of self-care monitoring on autonomous and consulting self-care management behaviors are shown to occur via item #14 (symptom recognition) in a sample of patients from the United States, Italy, and Sweden (*n* = 1615). Note that the dashed arrow indicates the direct effects were not statistically significant. The error standard is shown in brackets. *Key:* SC-CII: Self-Care of Chronic Illness Inventory; a: *p*-value 0.05; b: *p*-value < 0.05; US, United States; ITA, Italy; SWE, Sweden.

The goodness of fit statistics for the mediation model were as follows: $\chi_{\left(163,\text{N}=1,615\right)}^{2}$ = 272.163, *p* < 0.001, CFI = 0.969, TLI = 0.962, RMSEA = 0.035 (90% CI = 0.028 0.043), *p* = 1.000, SRMR = 0.055. Then we tested a mediation model where we posed equality constraints on the unstandardized betas related to direct paths linking the model's constructs. All constraints posed on these direct effects (symptom recognition (item #14) on consulting and autonomous behaviors, Self-Care Monitoring on symptom recognition, consulting, and autonomous behaviors) among the US, Italian, and Swedish samples were tenable. The results of specific indirect effects and the direct effects of the model mediation among the three countries are presented in the second panel of Table 1 and Figure 3, respectively. Briefly, symptom recognition (measured by item #14) mediated the relationship between Self-Care Monitoring and autonomous behaviors of Self-Care Management (β = 0.098, β = 0.122, β = 0.081, *p* < 0.001) but not of consulting behaviors (β = −0.009, β = −0.008, β = −0.007, *p* > 0.05) for US, Italy and Sweden, respectively. In addition, all effects tested were invariant excepted for the effect of Self-Care Monitoring on item #14 among the US, Italian, and Swedish samples. Since both direct and indirect effects were significant in this model, we conclude that *partial* mediation was found in the three countries.

Figure 3 shows the results of a multiple-group analysis. This approach allowed us to simultaneously test a unique model on two or more samples maintaining the specificity of each sample. Equality constraints allow testing for the invariance/equivalence of a same parameter (e.g., a regression coefficient linking two focal constructs) across samples. In a single sample analysis data are aggregated in a single dataset, thus the specificities of the different samples get blurred. Moreover, in our case, when combining datasets from the United States (*n* = 407), Italy (*n* = 784) and Sweden (*n* = 438) into a single sample would risk getting a solution where the largest sample (Italy) would influence the results more than the smaller samples. Results of the multiple-group analysis shows clearly that all equality constraints except one are tenable: we also included in the revised (Figure 3) the non-standardized estimates, from which it is evident that estimates for the constrained parameters are the same across countries.

## Discussion

The aim of this study was to test the hypothesis that symptom recognition is a mediator of the relationship between self-care monitoring and self-care management. In addition to finding a direct relationship between these two theoretical concepts, symptom recognition mediated the relationship between self-care monitoring and the autonomous behavior dimension of the Self-Care Management scale. These findings add significantly to the Theory of Self-care of Chronic Illness (10) by helping to clarify the role of symptom recognition in the self-care behavioral process. It appears that patients who recognize their symptoms are more likely to engage in autonomous self-care, those behaviors that can be initiated and performed by patients in response to symptoms (e.g., rest). However, symptom recognition does not necessarily influence consulting self-care management behaviors. That is, those who struggle with symptom recognition appear to move directly from self-care monitoring to consulting with a healthcare provider. Note that we do not know at this point if this is a characteristic of a patient who never recognizes symptoms or if this specific symptom was unrecognized but another one was recognized. Further research is needed to clarify the role of symptom recognition. If confirmed in other contexts and in longitudinal analysis, this new understanding of symptom recognition will have important implications for clinical practice, instrument refinement, and future theory and research.

A variety of subtly different processes are involved in symptom recognition, which has made the literature on the topic confusing. Posey (35) defined symptom perception as a belief held by the person experiencing the symptom about what it means (cognitively and emotionally), which emphasizes recognition or interpretation of the event. A recent qualitative study describes symptom monitoring, awareness, and evaluation as symptom perception processes (36). These investigators found that symptom monitoring promoted detection, but active monitoring was not essential because sudden changes were noticed regardless of routine monitoring. They also noted that becoming aware of symptoms promoted symptom monitoring, creating a feedback loop between symptom monitoring and awareness. Another study found two patterns of symptom recognition in patients with an acute exacerbation of chronic obstructive pulmonary disease (37). Symptom recognition involved meaning, patterns, warning signs, prodromal symptoms, and risk factors. Symptom recognition influenced the self-care behaviors of study participants. Physiological factors were cited as causing symptom exacerbation but elements of interoception were not identified.

Although self-care is a behavioral process, symptom recognition is not a behavior. Instead, symptom recognition involves cognitive, affective, and physical factors that *influence* behavior. The interpretation of a symptom adds reasoning (35) and stimulates a judgment, a decision, and a behavioral response (38). Symptoms can be vague, variable, and misconstrued by patients as related to another illness or the aging process (1, 9, 12). Certain people are relatively more likely to detect and consciously perceive symptoms. Stress and depression influence the response to symptoms (39, 40). In a prior study, an improvement in the symptoms of depression was significantly associated with improvements in autonomous self-care behaviors (41). Those with neurotic personality characteristics (i.e., pervasive negative way of viewing the world, self-consciousness, and concern with bodily processes), and those in boring situations without distractions (42) may have better recognition of symptoms. Culture influences what people think about symptoms, with causal attributions reflecting efforts to make sense of physical and emotional experiences (43–45). These cognitive and affective factors have been studied widely to understand the nature of symptom recognition (46), but physiological factors are less well studied.

The results of this study support analyzing the item assessing symptom recognition as a single item separately from the scale scores now used in the SC-CII. Future research is needed to develop a full self-report scale measuring symptom recognition; a scale composed of more than a single item would increase the reliability with which the construct is measured and eventually allow investigators to consider different aspects of symptom recognition. For now, we recommend a revised scoring procedure that involves analyzing item #14 (*The last time you had symptoms, how quickly did you recognize it as a symptom of your illness?*) as a single item using descriptive statistics. The item is probably most informative when analyzed as the percentage of study participants who have not had symptoms, did not recognize the symptom when it occurred, or did not recognize it quickly, etc. Scoring of the Self-Care Maintenance scale remains unaffected. The Self-Care Monitoring scale should be tallied as a combination of items 9–13 and then standardized 0–100. The Self-Care Management scale should be tallied as a combination of items 15–20 and then standardized 0–100. By scoring item #14 on symptom recognition separately from the other three SC-CII scales, further research may help us to understand the specific contribution of symptom recognition to self-care.

Future research may support considering symptom recognition as a separate concept in the Theory of Self-Care of Chronic Illness (10). We also recommend that researchers consider testing symptom recognition as a moderator of self-care management. It may be that individuals who are unable to recognize their symptoms are those who are poorest in self-care management. Such a finding might encourage investigators to change their approach to improving self-care management by focusing on compensating for poor symptom recognition rather than addressing it as a modifiable behavior. For example, if poor symptom recognition is related to a neural defect or depression, it may be impractical to focus on patient education. Instead, the use of tele-monitoring technology and decision support using artificial intelligence may offer opportunities to target specific challenges in symptom recognition.

In the future, we will consider whether the recommended scoring approach should be used with our other self-care measures (https://self-care-measures.com/). The item measuring symptom recognition is used in the Self-Care of Heart Failure Index (47), the Self-Care in Chronic Obstructive Pulmonary Disease Inventory (48), and in the Self-Care of Diabetes Inventory (49), as well as in the Caregiver Contribution to Self-Care of Heart Failure Index (50). We will continue to study how this item functions in ongoing studies. A deeper theoretical reflection may support a decision to revise the scoring in these instruments as well.

### Limitations

Even though we enrolled a large sample of participants from three countries, we used convenience sampling to select patients in the US and Italy (the Swedish sample was consecutive). We performed a secondary analysis of data that were collected for other purposes. The data used are cross-sectional, so strong caution should be used in deriving a causal interpretation of the influence paths linking the variables. No specific validity testing was performed on item #14 as a measure of symptom recognition. Consequently, our finding should be generalized with caution.

## Conclusion

These results support the growing body of research on interoception as a physiologic phenomenon influencing symptom perception. Investigators are encouraged to reflect further on the reasons for poor symptom recognition in their patient populations and continue to explore symptom recognition as a bridge between self-care monitoring and self-care management in future studies. Further, as symptom recognition alone is insufficient to fully capture issues such as treatment seeking delay, we encourage investigators to evaluate decision-making, conflict resolution, and memory to make the symptom recognition question more interpretable. This analysis also supports a change in scoring of the SC-CII. Investigators are encouraged to use the item asking about symptom recognition alone rather than using it as part of either the Self-Care Monitoring or the Self-Care Management scale.

## Data Availability Statement

The original contributions presented in the study are included in the article/supplementary materials, further inquiries can be directed to the corresponding author.

## Ethics Statement

The studies involving human participants were reviewed and approved by University of Pennsylvania, University of Rome Tor Vergata, Linkoping University. The patients/participants provided their written informed consent to participate in this study.

## Author Contributions

All authors listed have made a substantial, direct, and intellectual contribution to the work and approved it for publication.

## Conflict of Interest

The authors declare that the research was conducted in the absence of any commercial or financial relationships that could be construed as a potential conflict of interest.

## Publisher's Note

All claims expressed in this article are solely those of the authors and do not necessarily represent those of their affiliated organizations, or those of the publisher, the editors and the reviewers. Any product that may be evaluated in this article, or claim that may be made by its manufacturer, is not guaranteed or endorsed by the publisher.
